# Objectively measured physical activity levels and sedentary time in children and adolescents with sickle cell anemia

**DOI:** 10.1371/journal.pone.0208916

**Published:** 2018-12-06

**Authors:** Hugo Nivaldo Melo, Simone Joanna-Maria Stoots, Marijn Aimee Pool, Vitor Oliveira Carvalho, Max Luan De Carvalho Aragão, Ricardo Queiroz Gurgel, Charles Agyemang, Rosana Cipolotti

**Affiliations:** 1 Federal University of Sergipe, Aracaju, Brazil; 2 University of Amsterdam, Amsterdam, Netherlands; 3 Department of Public Health, Academic Medical Centre, University of Amsterdam, Amsterdam, Netherlands; Université Claude Bernard Lyon 1, FRANCE

## Abstract

The aim of this study was to identify the levels of physical activity and sedentary behaviour of children and adolescents with sickle cell disease (SCA) compared to healthy individuals. A cross-sectional study with a quantitative approach was performed at a reference center for the treatment of patients with hemoglobinopathies in northeastern Brazil. Patients were recruited between October 2015 and January 2017. Eligible participants answered a Physical Activity Questionnaire for Older Children and Adolescents (PAQ-C) and were instructed to use an ActiGraph wGT3X-BT triaxial accelerometer for seven consecutive days. Fifty patients (and their 50 controls matched for age and sex) were then evaluated. We observed lower moderate (19.2 ± 11.9 and 27.1 ± 13.8 min/d; p<0.01) and vigorous PA (3.6 ± 4.1 and 7.8 ± 7.4 min/d; p<0.01) in cases than controls, respectively. There was also a significant difference among cases and controls in the following variables: total of steps (51010 ± 19600 and 59105 ± 22650; p = 0.04) and “total caloric expenditure” (1015 ± 516 and 2404 ± 1308; p<0.01), with the lowest values for the patients with SCA for all variables. Children and adolescents with SCA presented lower levels of physical activity than healthy children and adolescents, either when evaluated by PAQs or by accelerometer.

## Introduction

Sickle cell anemia (SCA) is a neglected tropical disease [1] characterized by a point mutation in the β-chain hemoglobin (Hb) gene. When deoxygenated, HbS, the Hb resulting from the mutation, polymerises, resulting in a change in the red blood cells and assuming a sickle shape. The falcized red blood cells can obstruct the microcirculation, resulting in ischemia-reperfusion injury, with inflammatory cytokines, pain and functional impairment, particularly in the musculoskeletal system [2], which can lead to defensive sedentary behaviour even among young patients.

Previously, it has been demonstrated that childhood physical activity (PA) has beneficial effects on health, both in the short and long terms, and may reduce risk factors for chronic diseases [3]. More recently, one study concluded that moderate physical exercise is not harmful for patients with SCA [4], and, in an animal model (rats with SCA), it is suggested that PA could be beneficial in the clinical course of the disease [5].

The evaluation of PA is complex due to its multi-dimensional characteristics. Questionnaires that use semi-quantitative scales, such as the Physical Activity Questionnaire for Older Children and Adolescents (PAQ-C), have the advantage of easier applicability, but they are influenced by the interviewee's perception of level of PA [6]. Thus, objective methods are preferable and accelerometer is an instrument that generates quantitative results and thus confers objectivity to the assessment of PA in children and adolescents [7].

Thus, this study aimed to evaluate objectively and subjectively the level of PA and sedentary behaviour of children and adolescents with SCA compared to healthy individuals by triaxial accelerometer and questionnaire, respectively.

## Methods

### Design

This is a cross-sectional study carried out at the outpatient clinic of a university center in the northeast of Brazil, which is a regional reference for the treatment of patients with SCD. After initial screening, eligible patients and healthy controls completed PAQ-C [6,8]. Subsequently, participants and their caregivers were instructed on how to properly use the ActiGraph wGT3X-BT triaxial accelerometer and asked to use it for seven consecutive days.

### Population

We accessed a registry of patients with SCA who regularly attended the outpatients’ clinic in the study institution (352 children and adolescents). Two hundred eighty-eight patients did not meet the inclusion criteria. Sixty-four patients were considered eligible, but 14 were later excluded because they did not use the accelerometer for seven consecutive days. Patients were recruited from October 2015 to January 2017. Among the patients with SCA (homozygous for HbS) confirmed by hemoglobin electrophoresis, individuals from 6 to 18 years-old in stable clinical condition were considered eligible, if they had not received blood transfusions during the last three months and if they had not any acute complications for at least one month. Patients with neurological or orthopedic impairment were excluded.

The control group consisted of healthy children and adolescents recruited at a local public school and matched for age and sex with the patients.

### Ethics statement

This study was approved by the Research Ethics Committee involving Human Beings of the Federal University of Sergipe (protocol: 30661314.0.0000.5546). All guardians responsible for the patients and controls signed a free and informed consent form.

### Laboratory tests

The following exams were performed in SCA patients: hemogram (automated, Cell Abnormal Rubber Analyzer, Abott®), which provided quantification of hemoglobin, hematocrit, erythrocyte counts, mean corpuscular volume, leukocytes, neutrophils and platelets; reticulocyte count (bright cresyl blue method); Indirect bilirubin dosage (diazoreagent method, Wiener-lab kit); Lactate dehydrogenase (Automated Kinetic Lactate Oxidase Method, Wiener-lab kit); quantification of hemoglobin S (Isoelectric focusing method).

All laboratory analysis were performed under stable clinical conditions within four weeks prior to the application of the accelerometer.

### Medication use

All patients were taking folic acid supplements (2 mg/d). Twenty-four patients were receiving hydroxyurea. These patients started at 15 mg/kg/d and were currently receiving the standard dose (20 to 35 mg/kg/d)[9] for at least 12 months.

### Physical activity questionnaire for older children and adolescents (PAQ-C)

All patients included in this study completed the Brazilian version of PAQ-C [8], composed by nine questions about sports, games and other physical activities at school and at leisure activities. This questionnaire aims to provide a complete picture of the type and amount of PA that the participant had been performing in the last seven days prior to the interview. Each question was scored on a scale of 1 to 5, being: very sedentary (1), sedentary (2), moderately active (3), active (4) or very active (5). In order to determine the final score, the mean of all responses was calculated.

### PA measurements

The ActiGraph GT3X Accelerometer (ActiGraph LLC, Pensacola, FL, USA) was used to objectively monitor the time spent in PA and sedentary behaviour. The accelerometer was utilized on an elastic belt and participants were instructed to position it on the hip line of their dominant side. Participants used the device for 7 consecutive days, including two weekend days for at least 10 hours a day [10,11].

The study team instructed and monitored the children and their caregivers to remove the monitor during aquatic activities and during sleep. The accelerometer was initialised by the researcher responsible for the study through the manufacturer's software (ActiLife version 6). We analyzed all three axes (x, y and z) vector magnitude (Vm) activity counts, calculated as: Vm = √ (X2 + Y2 + Z2). In order to record the movement in counts per minute, the count was set to 60 second epochs.

Sedentary behaviour period was defined by <100 count per minute [12,13]. Values ​​between 100 and 1999 counts per minute were recorded as light PA (LPA) [13]. The time spent on moderate PA (MPA) and vigorous PA (VPA) was calculated based on cutoffs of 2000 and 4000 counts per minute, respectively [14]. The PA of each individual was categorised in the three intensity levels (LPA, MPA and VPA) and the average daily sedentary time had been recorded. The time spent in moderate/vigorous PA (MVPA) was calculated as the sum of MPA and VPA.

Resting energy expenditure was estimated from age specific prediction equations to derive the metabolic equivalent of MET intensity levels. The equation was: METs = 2.757 + (0.0015 * counts per minute)–(0.08957 * age (yr))–(0.000038 * counts per minute * age (yr)) [7].

The daily percentage of all PA intensity levels was calculated based on the time spent at each intensity level, including sedentary time [15]. For comparison, the children were considered to be in accordance with the recommendations of the PA when the mean MPVA over all measured days was 60 min or more [16]. The mean time measured for both weekdays and weekends was calculated by summarising the sedentary time and the time spent at different PA intensities.

### Statistical analysis

The data analysis was performed using SPSS version 13.0 for Windows (SPSS, Inc., Chicago, IL, USA). Quantitative variables were described as means, median and standard deviations. All variables were checked for normality prior to analysis using the Kolmogorov-Smirnov test. Differences between means were analysed using the Mann-Whitney U test and the chi-square test was used to evaluate the proportions of occurrence of categorical variables, comparing patient and control groups. Differences in time spent at different PA intensities and the mean times measured on weekdays and weekends were analysed by using the t-test for paired samples. The chi-square test was used to determine differences in the percentage of time spent at different PA intensities. The significance level used was 5% (p<0.05).

## Results

Fifty patients were included in this study, of which 60% were male with a mean age of 12.02 ± 3.63 years.

Considering the mean value of hemoglobin in the group of patients, they were categorized into two groups (Hb ≥8 and Hb <8). Associations between Hb category and the variables obtained from the accelerometer were tested. It was observed that the total number of minutes spent in vigorous activity was significantly lower in the group with Hb <8 (p = 0.03). There was no association for the other variables studied.

All 50 patients and 50 controls used the accelerometer for seven consecutive days without any complications. The clinical characteristics of both groups (patients with SCA and healthy controls) are described in Table 1.

**Table 1:** Characterization of study participants.

Variables	SCA children and adolescents (n = 50)	Range	Controls (n = 50)	Range	p
Height (m)	1.41 ± 0.17	1.13–1.77	1.47 ± 0.16	1.24–1.81	0.09
Weight (kg)	34.2 ± 12.9	17.2–66.1	47.2 ± 16.4	20.7–78.2	**<0.01**
BMI (kg/m^2^)	16.3 ± 2.4	13.0–23.4	21. ± 4.5	14.3–30.2	**<0.01**
Age (yrs)	12 ± 3.6	6–18	11 ± 3.4	6–18	0.14
Sex male (%)	60	-	58	-	
Hb (g/100mL)	8.12 ± 1.16	5.03–12.15	-		
Hematocrit (%)	22.4 ± 3.8	14.2–38.1	-		
RBC (10^1^^2^/L)	2.46 ± 0.72	1.66–5.18	-		
Platelets (10^9^/L)	419 ± 123	109–878	-		
Leukocytes (10^9^/L)	11.7 ± 2.9	4.1–20.7	-		
Neutrophils (%)	47.7 ± 8.9	30–78	-		
Reticulocytes (%)	9.15 ± 4.79	07–19.21	-		
Indirect bilirubin (mg/dL)	2.82 ± 2.76	03–17.09	-		
MCV (fL)	86 ± 17	31.07–125	-		
LDH (U/L)	957 ± 480	100–2127	-		
Hydroxyurea therapy (HU) (number of patients, %)	24; 48	-	-		
HBS (%)*	74.3 ± 14.2	34.4–95	-		

Results are expressed as mean, standard deviation and range unless otherwise indicated. *BMI*: Body mass index, *Hb*: hemoglobin, *RBC*: Red blood cells, *MCV*: Mean corpuscular volume, *LDH*: Lactate Dehyrogenase, *HbF*: Fetal hemoglobin, *HbS*: hemoglobin S. * the results include four patients with hereditary persistence of HbF (HPFH).

The groups were similar in terms of age and distribution by sex, meeting the pairing criteria, but presented a difference in the means of body mass and body mass index (BMI).

There was a statistically significant difference between the groups in the variables "daily mean of moderate and vigorous physical activity" (p<0.01) with patients performing worse than the control group (Table 2).

**Table 2:** Physical activity and sedentary behavior variables (data from ActiGraph).

Variables	SCA children and adolescents (n = 50)	Median/Range	Controls (n = 50)	Median/Range	p
Vector Magnitude Counts (KS: p = 0.2)	3967142 ± 1743719	3750275/ 1263797–8678018	5194904 ± 2098832	5391045/ 1382923–9690246	**<0.01**^**T**^
Vector Magnitude CPM (KS: p<0.001)	764 ± 297	671/ 311–1511	1096 ± 1096	1017/ 253–8 8360	**<0.01**^**W**^
Sedentary Time (min/day) (KS: p = 0.2)	416.8 ± 106	412/ 158.8–651	409.3 ± 114.3	381/ 170.3–835.2	0.63^T^
Sedentary Time (%) (KS: p = 0.2)	56.8 ± 13.9	57/ 30.6–95.5	52.5 ± 11.7	50/ 32.7–77.4	0.45
Light PA (min/day) (KS: p = 0.2)	325.5 ± 114.1	317/ 92.5–651	337.1 ± 93.9	355/ 110–439.6	0.23^T^
Light PA (%) (KS: p = 0.2)	42.9 ± 10.7	40/ 22.9–95.5	47.7 ± 14.8	45/ 20.1–59.7	0.42
Moderate PA (min/day) (KS: p = 0.01)	19.2 ± 11.9	14/ 1.8–59.6	27.1 ± 13.8	24/ 0.7–62.2	**<0.01**^**W**^
Moderate PA (%) (KS: p = 0.2)	2.6 ± 1.8	2/ 0.3–8.3	3.5 ± 1.9	3/ 0.1–8.3	0.36
Vigorous PA (min/day) (KS: p<0.001)	3.6 ± 4.1	2/ 0–25.2	7.8 ± 7.4	6/ 0–46.2	**<0.01**^**W**^
Vigorous PA (%) (KS: p<0.001)	0.5 ± 0.6	0.2/ 0–3.5	1.1 ± 0.9	0.7 0–5	0.35
MPVA (min/day) (KS: p<0.001)	22.9 ± 18.6	32/ 3.7–94	35 ± 66.6	33/ 2.5–306.2	0.70^W^
MPVA (%) (KS: p<0.001)	3.1 ± 4.3	4.5/ 0.4–9.1	4.6 ± 2.4	4.6/ 0.5–11.2	0.42
MET (KS: p<0.001)	1.71 ± 0.4	1.6/ 1.17–2.45	1.87 ± 0.39	1.9/ 1.12–2.57	**0.04**^**W**^
Current PA recommendations (%)	8	-	30	-	
Kcal total (KS: p<0.001)	1015 ± 516	862/ 701–2659	2404 ± 1308	2263/ 806–5178	**<0.01**^**W**^
Sitting (%) (KS: p = 0.01)	18.3 ± 5.1	18/ 7–32	19.1 ± 5.8	18/ 11–36	0.15
Standing (%) (KS: p = 0.05)	24.9 ± 7.3	25/ 6–40	25.6 ± 7.4	27/ 10–39	0.68
Lying (%) (KS: p<0.001)	5.1 ± 3.8	4/ 0–19	6.2 ± 5.7	5.5/ 0–28	0.25
Total steps (KS: p = 0.2)	51010 ± 19600	51479/ 16057–120567	59105 ± 22650	59429/ 20583–137817	**0.04**^**T**^

Results are expressed as mean, standard deviation, median and range unless otherwise indicated. *KS*: Kolmogorov–Smirnov test, *CPM*: counts per minute, *PA*: physical activity, *MPVA*: moderate-to-vigorous physical activity, ^*W*^: Mann-Whitney U test, ^*T*^ t-test.

There was a statistically significant difference between groups in the "Metabolic Equivalent" (MET) variables, with p = 0.04, “Total Steps” (p = 0.04) and “total energy expenditure” (p<0.01), with the lowest values ​​always occurring in the patients’ group (Table 2). In the studied sample, 8% of patients with SCA and 30% of controls complied with the current recommendations of PA [16].

There was no statistically significant difference in the average time of sedentary activity (neither in total or in the daily average), but these values were always lower in the control group.

The questionnaire (PAQ-C) was applied to all participants. The mean score obtained by the patients was 1.65 ± 0.4 and the mean score by the controls was 3.39 ± 0.38. Sixty two percent of the patients with SCA were categorised as very sedentary and the remaining 38% were sedentary (Table 3). Among the controls, 11% were classified as sedentary, 75% as moderately active, and 14% were active. The rate of sedentary individuals was significantly higher among patients than controls (p<0.01).

**Table 3:** Comparison of physical activity levels in different categories assessed by the PAQ-C between patients and controls.

Variables	SCA children and adolescents (n = 50)	Range	Controls (n = 50)	Range	p
Spare-time activity	0.75 ± 0.31	0–1.6	2.48 ± 0.61	1.4–3.7	**<0.01**^**W**^
Activity during PEC	1.74 ± 0.62	1–3	3.36 ± 0.65	2–5	**<0.01**^**W**^
Break-time Activity	1.61 ± 0.69	1–3	3.64 ± 0.59	2–5	**<0.01**^**W**^
Lunch-time Activity	1.42 ± 0.53	1–3	3.32 ± 0.71	2–5	**<0.01**^**W**^
After school Activity	1.85 ± 0.98	1–4	3.38 ± 0.56	2–4	**<0.01**^**W**^
Evening Activity	1.77 ± 0.84	1–4	3.58 ± 0.73	2–5	**<0.01**^**W**^
Weekend Activity	2.08 ± 0.75	1–3	3.63 ± 0.78	2–5	**<0.01**^**W**^
AF during the last 7days	2.32 ± 0.81	1–4	3.72 ± 0.54	3–5	**<0.01**^**W**^
AF during each day last week	1.62 ± 0.53	0.1–3	3.37 ± 0.58	2.5–4.3	**<0.01**^**T**^
Score total	1.65 ± 0.41	0.8–2.31	3.39 ± 0.38	2.7–4.2	**<0.01**^**W**^

*PEC*: Physical Education Classes; *AF*: Activity frequency; Data expressed as mean ± standard deviation and range (minimum and maximum). Independent t-test and Mann-Whitney U test were used to compare the two groups when the variables presented parametric and non-parametric distribution, respectively, ^*W*^: Mann-Whitney U test, ^*T*^ t-test.

Table 3 presents the comparison of the PA level in the different activity categories evaluated by PAQ-C and shows that patients with SCA reported lower PA levels in all categories compared to healthy controls.

## Discussion

The present study aimed to identify the intensity profile of PA in children and adolescents with SCA, using an objective measurement tool (accelerometer) and subjective measure (questionnaire). The comparison between patients and their healthy controls evidenced the patients' sedentary profile and the difference in the level of PA intensity between the patients and the controls. Specifically, within the group of patients we observed an association between sedentary behavior at lower levels of Hb corroborating previous results [17]. These results are of particular importance when considering the beneficial effects on the oxidative stress damages, previously identified in a study which used an animal model [5]. The authors of that study proposed that an exercise program could be useful for controlling clinical complications due to SCA [5].

The effects of an exercise program applied to heterozygous carriers for HbS (sickle cell trait) were previously evaluated and a study reported beneficial results on endothelial function, including reduction of oxidative stress markers and antioxidant enhancement (increased activity and NO availability) [18]. There is evidence that sedentary behaviour is associated with adverse health effects in groups of individuals with various chronic diseases [19], but patients with SCA have never been evaluated until now.

Impairment of nutritional status and growth retardation in children and adolescents with SCA are associated with resting energy expenditure 10–20% higher than that observed in healthy individuals, which is at least partially due to the higher cardiac output, such as mechanisms of compensation for moderate or severe and chronic anemia [20]. The present study showed a statistically significant difference in the MET variable, which reinforces the findings of a previous study [21].

Previous studies have evaluated the activity of the autonomic nervous system in patients with SCA and have identified an imbalance caused by parasympathetic activity at rest [22] and deficiency of autonomic reactivity [23]. Furthermore, the degree of impairment is associated with clinical severity [24]. The present study did not evaluate the activity of the autonomic nervous system. However, in healthy individuals, the energy expended with PA is positively associated with the activity of the autonomic nervous system, especially the activity of the parasympathetic nervous system [25]. Regular PA increases the activity of the parasympathetic nervous system, which has a protective effect on the cardiovascular system [26], whilst sedentary behaviour leads to an imbalance in the autonomic nervous system activity, which may favour the development of cardiovascular diseases [27], a condition that is particularly detrimental to the patients with SCA.

The present study identified low PA levels and low energy expenditure in patients with SCA compared with healthy individuals, corroborating previous studies [5,19,28,29]. Various factors, such as muscular hypotrophy, pulmonary and cardiac complications, may explain these findings [5].

However, intense physical exercise induces metabolic and physiological changes that may be detrimental to individuals with SCA [5] and there is no consensus on the maximum intensity of safe exercise that these patients can tolerate. In addition, due to the limitations imposed by the disease and its frequent acute intercurrences, parents of children and adolescents with SCA may discourage them from engaging in physical activities [30], which may explain the low energy expenditure and physical activity in the sample studied.

A previous study identified positive effects in MVPA for patients with SCA [31] and considered this practice to be safe. Considering the findings of this study, future objectives are to identify which training modalities would be better tolerated and could provide the greatest health benefits to patients. Given the findings, it is suggested that the evaluation of PA should be part of the outpatient follow-up for patients with SCA, being an important tool to determine the severity of the disease and to suggest a possible strategy to prevent clinical complications.

We identified a limitation of the present study: the use of the accelerometer was voluntarily and in the absence of acute intercurrences. Thus, it is possible that patients with more severe forms of SCA have not been included. However, given the results obtained, it is assumed that the inclusion of patients with greater frequency or intensity of symptoms would result in less PA and a more sedentary lifestyle.

## Conclusion

Children and adolescents with SCA were assessed for PA, assessed subjectively by the PAC-C and objectively by the accelerometer, resulting in values lower than that of healthy children and adolescents.

## Supporting information

S1 File Database of sedentary behavior and level of physical activity in patients with sickle cell disease and healthy controls. (XLS)
